# Fluid Shifts and Muscle Loss in Critical Care: Accuracy of Ultrasound Versus Bioelectrical Impedance Analysis

**DOI:** 10.3390/nu18122019

**Published:** 2026-06-21

**Authors:** Gintarė Šostakaitė, Martyna Jauniškytė, Dominykas Budrys, Kastytis Budrevičius, Erika Šalčiūtė-Šimėnė, Marija Svetikienė, Tomas Jovaiša, Tadas Žvirblis, Andrius Klimašauskas, Jūratė Šipylaitė

**Affiliations:** 1 Clinic of Anaesthesiology and Intensive Care, Faculty of Medicine, Vilnius University, LT-01513 Vilnius, Lithuaniatomas.jovaisa@santa.lt (T.J.);; 2 Faculty of Medicine, Vilnius University, LT-01513 Vilnius, Lithuania; 3 Institute of Data Science and Digital Technologies, Faculty of Mathematics and Informatics, Vilnius University, LT-01513 Vilnius, Lithuania

**Keywords:** muscle ultrasound, bioelectrical impedance analysis, intensive care unit, fluid balance, muscle wasting, systemic inflammation

## Abstract

**Background**: Critically ill patients experience rapid muscle wasting during their ICU stay. Ultrasound (US) and bioelectrical impedance analysis (BIA) are widely used to assess muscle mass; however, their accuracy may be affected by fluid balance alterations. This study aimed to compare the reliability of US and BIA in detecting muscle loss under varying fluid balance conditions in ICU patients. **Methods**: In this prospective observational study, adult ICU patients with an ICU stay of ≥7 days were evaluated on Days 1, 5, and 7. Muscle thickness was measured using US, and phase angle (PhA) using BIA. Cumulative fluid balance, C-reactive protein (CRP), and lactate levels were recorded. Patients were stratified according to cumulative fluid balance. **Results**: A total of 143 ICU patients were included in the final analysis. US demonstrated a progressive decrease in muscle thickness (−3.54% ± 10.90% from Day 1 to Day 5 and −7.56% ± 11.82% from Day 1 to Day 7 (both *p* < 0.0001)), whereas BIA showed no significant change in PhA. Positive fluid balance significantly reduced PhA compared with the negative balance group, *p* < 0.001, whereas no statistically significant effect on US measurements was detected. CRP > 200 mg/L was associated with greater US-detected muscle loss on Day 5, while lactate > 2.5 mmol/L was associated with lower PhA. **Conclusions:** Ultrasound reliably identified structural muscle wasting in critically ill patients, with no statistically significant effect of fluid balance detected in this cohort. Furthermore, ultrasound measurements were associated with inflammation-related muscle loss. In contrast, BIA was strongly influenced by hydration and perfusion status, limiting its ability to assess true muscle mass loss in the ICU setting.

## 1. Introduction

Patients in the ICU can lose up to 2% of their muscle mass daily, a decline associated with reduced muscle strength, functional capacity, and physical performance [1,2]. This contributes to prolonged ICU stays and higher short-term and long-term mortality [3,4]. Muscle wasting in the ICU is driven by several factors, particularly during the first week of treatment: the inflammatory response, immobility, malnutrition, and increased muscle protein breakdown due to critical illness [5]. Assessing muscle mass loss during an ICU stay presents a significant challenge for clinicians in intensive care. Methods used to evaluate muscle wasting in critically ill patients include computed tomography, dual-energy X-ray absorptiometry, whole-body magnetic resonance imaging, muscle US, and bioelectrical impedance analysis (BIA) [6]. Among these, dual-energy X-ray absorptiometry, isotope dilution studies, whole-body magnetic resonance imaging, and computed tomography are regarded as reference (gold-standard) techniques for body composition and muscle mass assessment, each capturing body composition at different levels (molecular vs. tissue–organ) [7]. However, they are not practical for use in critical care settings or at the bedside because they tend to be logistically demanding and time-consuming [8,9,10].

Under physiological conditions, water constitutes approximately 50% to 60% of total body mass and about 75% of muscle mass [11]. During active muscle contraction, intramuscular microcirculation decreases, but the water content remains unchanged; instead, there is a reduction in muscle volume due to fluid shifting into the extracellular space, as observed by measuring the intracellular water (ICW)/total body water (TBW) ratio and assessing muscle thickness via US [11,12,13]. In intensive care patients, the water content in muscle remains unchanged, while the proportions of intracellular and extracellular water [14]—as well as the muscle structure itself—undergoes alterations [15].

BIA and its most popular measurements—the PhA and fat-free mass (FFM)—though widely used for assessing body composition under normal conditions, have been proven to be inaccurate in ICU patients due to fluctuations in fluid balance, which can lead to misinterpretation of muscle status in this population [14]. Nevertheless, other BIA parameters, such as TBW, ICW, extracellular water (ECW), the ECW/ICW ratio, and the FFM hydration index (FFMH), are recommended for guiding fluid management and assessing the fluid status [16,17], and they may prove valuable in evaluating the impact of fluid status on muscle condition.

Research comparing BIA and muscle US across varying degrees of fluid balance in ICU settings is limited. Some studies have indicated that US might be more accurate than BIA for monitoring muscle mass [1,14]. However, it remains unclear whether and to what extent fluid balance affects US measurements, as it does with BIA. This study aims to address that gap by investigating the reliability of US in detecting muscle wasting, even in the presence of significant fluid shifts as assessed by BIA.

Compared with our previous publication, which primarily compared US and BIA for monitoring muscle mass loss during ICU stay, the present study provides an extended analysis with a larger cohort and a broader physiological focus. Additional patients were included, including patients with COVID-19 pneumonia, and the analysis was expanded to assess the influence of cumulative fluid balance, systemic inflammation, and tissue hypoperfusion on both US- and BIA-derived measurements. In addition to PhA and muscle thickness, we analysed additional BIA-derived parameters, including FFM, SMM, FFMH, ICW/TBW, and ECW/TBW. Therefore, the present study focuses not only on the agreement between US and BIA, but also on how fluid balance and acute physiological disturbances may modify the interpretation of bedside body-composition assessment in critically ill patients.

## 2. Materials and Methods

### 2.1. Study Design and Data Collection

This prospective study was conducted in two mixed ICUs at a tertiary teaching hospital in Lithuania (Vilnius University Hospital Santaros Klinikos). Eligible patients were adults (aged ≥18 years) with an ICU length of stay (LOS) of at least 7 days and a Sequential Organ Failure Assessment (SOFA) score of 3 or higher. The exclusion criteria were an ICU LOS of less than 4 days, the presence of a cardiac pacemaker, and limb amputation.

All patients were weighed using a bed weighing scale. For obese patients, the adjusted body weight (AjBW) was calculated (formula used for calculations: AjBW = IBW + 0.4 × (actual body weight − IBW)). The AjBW was calculated in order to provide a more accurate estimation of energy and protein requirements in obese patients. Height was determined either by direct measurement with a tape measure while the patient was lying flat or, if that was not possible, based on recollection by the patient or a family member.

Data collected included demographic information, comorbidities, admission diagnoses, APACHE II and SOFA scores, malnutrition risk score (NRS 2002), levels of sedation, muscle paralysis, fluid balance, levels of C-reactive protein (CRP), procalcitonin, peak lactate concentration, and albumin, as well as nutritional intake (calories and protein). ICU outcomes, including length of stay (LOS), duration of mechanical ventilation, and ICU survival, were also recorded. The primary outcomes were longitudinal changes in PhA (measured by BIA) and limb muscle thickness (measured by US). Additionally, exploratory analyses were performed to assess the influence of fluid balance, systemic inflammation (CRP), and hypoperfusion (lactate) on the accuracy of BIA and US measurements.

The methodology used in this study was based on our previously published work involving non-COVID-19 ICU patients [18]. The present manuscript represents an extended analysis of this research cohort. Compared with the previously published study, additional patients were included, COVID-19 patients were incorporated, and the analysis was expanded to evaluate the effects of cumulative fluid balance, CRP, lactate, and additional BIA-derived parameters on the interpretation of US and BIA measurements. The primary focus of the present study was not only to compare US and BIA, but also to determine how fluid balance, inflammation, and hypoperfusion may influence these bedside methods. This study received ethical approval from the regional bioethics committee (Research No. 158200-17-954-459), and informed consent was obtained from each patient or their legally authorized representative.

During BIA and US assessments, patients were positioned supine for 10 min, with arms abducted at a 15° angle from the trunk and legs positioned shoulder-width apart. Measurements of BIA, US, and fluid balance were taken on Day 1 (within 24 h of ICU admission), Day 5, and Day 7. All measurements were performed by a single investigator.

### 2.2. BIA

BIA quantifies and estimates body composition. When alternating current flows through the body, healthy cell membranes—acting as capacitors—delay the current by storing electrical energy. This delay creates a phase shift between current and voltage, measured as the PhA. The PhA reflects cellular health, particularly cell membrane integrity and function, and is a recognized marker of nutritional status and malnutrition. In BIA, measurements yield not only the PhA and skeletal muscle mass (SMM), but also estimates of ICW, ECW, and TBW. The ICW/TBW ratio, typically around 0.67, represents the proportion of TBW residing within cells and serves as an indicator of cellular mass and integrity. In healthy individuals, approximately two-thirds of TBW is intracellular; therefore, a reduced ICW/TBW ratio may suggest cellular dehydration or diminished cell mass. Conversely, the ECW/TBW ratio, normally about 0.33, reflects the proportion of water in the extracellular compartment; an elevated ratio may indicate fluid accumulation or inflammation. Additionally, the FFMH, calculated as (TBW/FFM × 100%), quantifies the percentage of FFM composed of water, thereby reflecting the hydration status of lean tissue. In healthy populations, FFMH values typically range from 73% to 75%, with deviations potentially indicating dehydration, fluid overload, or changes in body composition [19].

In this study, BIA measurements were conducted using the InBody S10 device (Biospace, Seoul, Republic of Korea), a single-frequency (50 kHz), phase-sensitive BIA device with a constant current of 400 μA. Contact electrodes were placed on all four limbs according to the manufacturer’s protocol (on both thumbs and middle fingers, and between the anklebones and heels). The PhA was recorded in degrees, the ICW/TBW and ECW/TBW ratios were calculated, and the FFMH was expressed as a percentage. Percentage changes in the PhA and FFMH from baseline (Day 1) to Day 5 and Day 7 were analysed. The ICW/TBW and ECW/TBW ratios were assessed on Day 1, Day 5, and Day 7.

### 2.3. US

Muscle US is a non-invasive imaging technique that uses ultrasound waves to visualise muscle structure and assess muscle health. Widely employed in clinical settings, US is used to evaluate the effects of injury, inflammation, and other conditions on muscle tissue.

In this study, muscle US was performed using a GE LOGIQ P9 machine (GE HealthCare Technologies Inc., Chicago, IL, USA) equipped with a 10 to 12 MHz multi-frequency linear array probe, set to musculoskeletal mode. A water-soluble transmission gel was applied, and no compression was used. Muscle thickness (in cm) was measured using electronic callipers on transverse images, targeting the biceps brachii (arm supinated, two-thirds of the distance between the acromion and cubital fossa) and the rectus femoris and vastus intermedius muscles (leg extended, midpoint between the anterior superior iliac spine and patella). To reduce measurement variability, all ultrasound measurements were performed by the same trained investigator using a standardized protocol. Each muscle was measured three times at each time point, and the mean value was used for analysis. Measurement sites were marked on the skin with a skin marker to ensure consistency across repeated assessments. Formal intra-rater reliability indices, such as intraclass correlation coefficient or coefficient of variation, were not calculated in the present cohort; however, the use of repeated measurements and a single trained evaluator was intended to minimize measurement error. Baseline muscle thickness was recorded on Day 1, with subsequent measurements on Days 5 and 7 expressed as a percentage of baseline. For each patient, measurements of the biceps brachii, rectus femoris, and vastus intermedius were performed bilaterally, and the average of all recorded values was used to calculate the percentage change in muscle thickness from baseline.

Both US and BIA measurements were conducted on all included patients at each designated time point.

### 2.4. Fluid Balance

Fluid balance was tracked throughout the ICU stay, given its potential impact on muscle mass measurements. Fluid intake included intravenous solutions and oral fluids (if patient was able to drink), medications, blood products, and enteral and parenteral nutrition, while fluid output accounted for urine, blood loss, drainage, gastric aspiration, and ultrafiltration during renal replacement therapy. Insensible losses were not estimated. The cumulative fluid balance was calculated by summing the daily fluid balances on Day 5 and Day 7.

### 2.5. Fluid Balance, CRP, and Lactate Categorization

For further subgroup analyses, patients were stratified into clinically relevant categories of fluid balance, CRP, and lactate levels (on Day 5 and Day 7). Fluid balance was categorized as negative (<−1000 mL), neutral (−1000 to 1000 mL), moderately positive (1000 to 3000 mL), and markedly positive (>3000 mL). CRP concentrations were divided into three categories (<100 mg/L, 100–200 mg/L, and >200 mg/L) to represent lower, intermediate, and higher systemic inflammatory burden. Lactate concentrations were categorized as <1.5 mmol/L, 1.5–2.5 mmol/L, and >2.5 mmol/L to distinguish patients with lower, moderately elevated, and higher lactate levels. These thresholds were defined a priori for exploratory subgroup analyses based on clinical interpretability and the need to maintain reasonably balanced subgroup sizes. They were not intended to represent validated cut-off values for predicting muscle wasting or for interpreting BIA and US measurements.

### 2.6. Statistics

Descriptive statistics, including frequency tables and mean with (standard deviation (SD)), were used to summarise quantitative and qualitative data, respectively. The paired-samples *t*-test was used to evaluate differences in parameters at consecutive time points. One-way analysis of variance was employed to evaluate mean differences when comparing three or more groups, with Bonferroni correction applied for pairwise comparisons. For the present analysis, univariate comparisons were performed to evaluate differences in PhA, BIA-derived parameters, and US-derived muscle thickness changes across fluid balance, CRP, and lactate categories. Multivariable linear regression models assessing predictors of PhA and muscle thickness changes were presented in our previous publication [18] and were not repeated in the present manuscript, as the focus of this analysis was the influence of fluid balance, inflammation, and lactate on the interpretation of US and BIA measurements. A two-tailed *p*-value of <0.05 was considered statistically significant. Statistical analysis was performed using RStudio version 2024.09.1.

Significant individual variance analysis was based on the level of change in both the PhA and US: delta PhA (ΔPhA) 1–5 and 1–7, and delta muscle thickness (ΔMTh) 1–5 and 1–7.

## 3. Results

**Figure 1:**
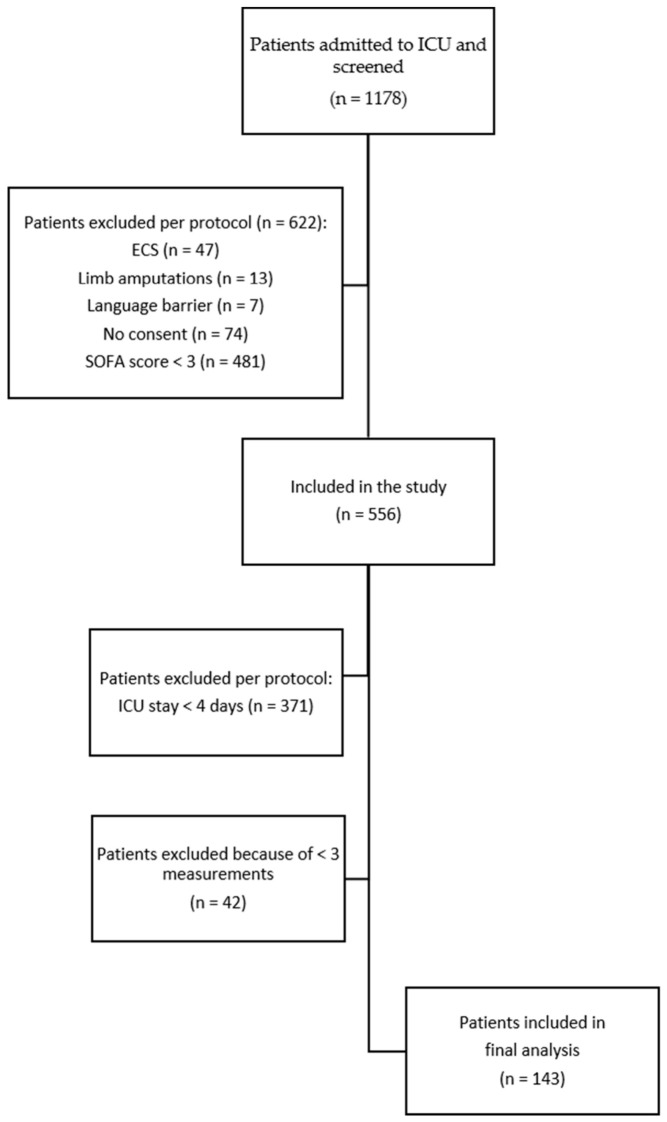
Patient inclusion flowchart.

In total, 1178 patients were screened, and 143 were enrolled in the study (Figure 1). The patients’ characteristics are presented in Table 1.

### 3.1. Relationship Between US Findings and PhA

US measurements showed a significant decrease in muscle thickness: −3.54% (10.9) between Days 1 and 5 and −7.56% (11.82) between Days 1 and 7 (*p* < 0.0001). By contrast, PhA measurements were −4.64% (17.56) and −4.56% (20.54), respectively, with no statistical significance.

The relative percentage change in muscle thickness (measured by US) was not correlated with the percentage change in PhA (measured by BIA) when comparing measurements from Day 1 to Day 5 (*p* = 0.154) or Day 1 to Day 7 (*p* = 0.121) of ICU treatment. A visual representation of these findings is provided in Supplementary Materials Figure S1.

### 3.2. Effect of Fluid Balance on Both Methods of Muscle Wasting Assessment

The patients were divided into four groups based on their accumulated fluid balance: negative (<−1000 mL), neutral (−1000 to 1000 mL), moderately positive (1000 to 3000 mL), and markedly positive (>3000 mL).

Patients with a positive fluid balance (moderately positive and markedly positive groups) demonstrated a significantly greater decrease in the PhA (−0.44° (0.796) and −0.47° (0.695)) between baseline and Day 5 compared with patients in the negative group (0.24° (0.714), *p* < 0.001). Similar results were observed between baseline and Day 7. No statistically significant effect of fluid balance category on US-derived muscle thickness changes was detected on either Day 5 or Day 7. However, this analysis was based on between-group comparisons and was not designed as an equivalence analysis; therefore, the absence of statistical significance should not be interpreted as proof of equivalence or complete absence of an effect. A detailed comparison is provided in Table 2 and Table 3.

### 3.3. Other Factors

**Figure 2:**
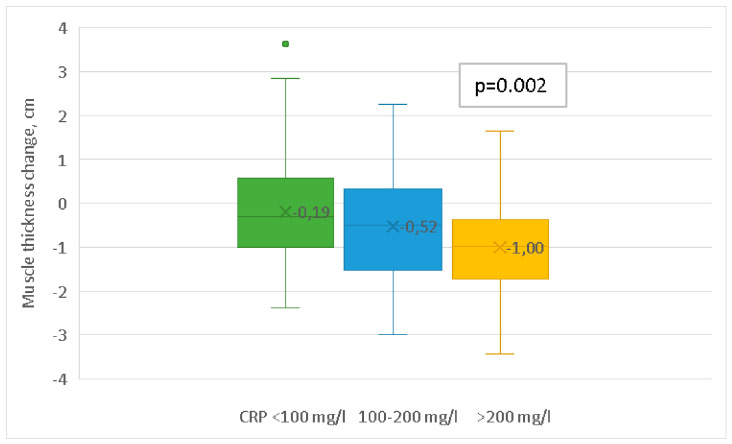
Change in muscle thickness (Day 5 vs. Day 1) stratified by CRP levels. Boxplot showing absolute values (cm) change in muscle thickness measured by US, grouped according to CRP levels on Day 5. A statistically significant difference was observed between the <100 mg/L and >200 mg/L CRP groups (*p* = 0.002).

**Figure 3:**
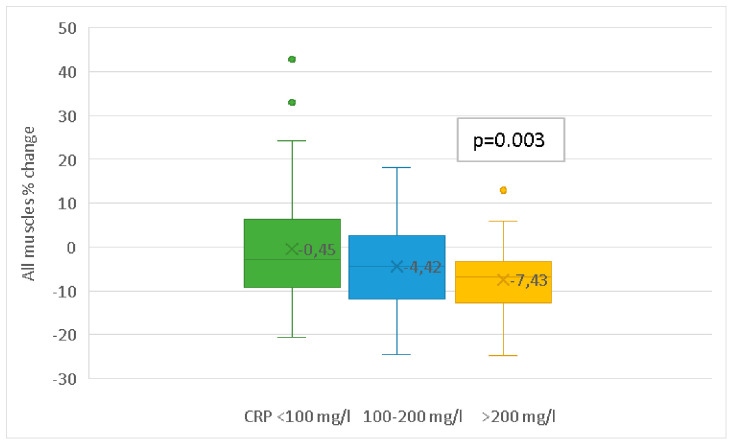
Percentage change in muscle thickness (Day 5 vs. Day 1) stratified by CRP levels. Boxplot showing percentage change in muscle thickness measured by US, grouped according to CRP levels on Day 5. A statistically significant difference was observed between the <100 mg/L and >200 mg/L CRP groups (*p* = 0.003).

We additionally evaluated the impact of inflammation on muscle loss. The patients were categorised into three groups based on the CRP concentration on Days 5 and 7: <100 mg/L, 100–200 mg/L, and >200 mg/L. Our findings showed that on both Day 5 and Day 7, BIA measurements (PhA, FFM, FFMH, and SMM, as well as ICW/TBW and ECW/TBW changes) did not differ significantly between CRP groups. However, higher CRP levels were significantly associated with more pronounced muscle mass loss: US measurements on Day 5 showed a statistically significant difference both in absolute values (*p* = 0.002) and in percentage change (*p* = 0.003) (Figure 2 and Figure 3). On Day 7, a statistically significant difference remained only for absolute muscle thickness measurements (*p* = 0.04) (Supplementary Materials Figures S2 and S3). Full data tables are provided in the Supplementary Materials Tables S1 and S2.

**Figure 4:**
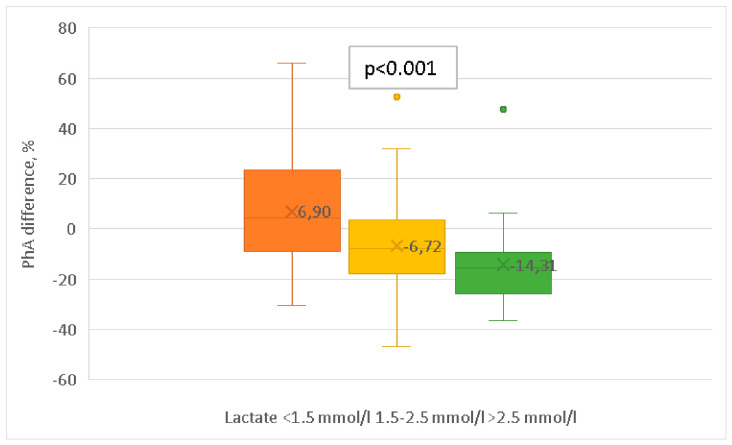
Percentage change in PhA (Day 7 vs. Day 1) stratified by lactate levels. Boxplot showing percentage change in PhA, measured by BIA, grouped by lactate levels on Day 7 in the ICU. Statistically significant differences were observed between the <1.5 and >2.5 mmol/L lactate groups, as well as between the 1.5–2.5 mmol/L and >2.5 mmol/L lactate groups (*p* < 0.001).

**Figure 5:**
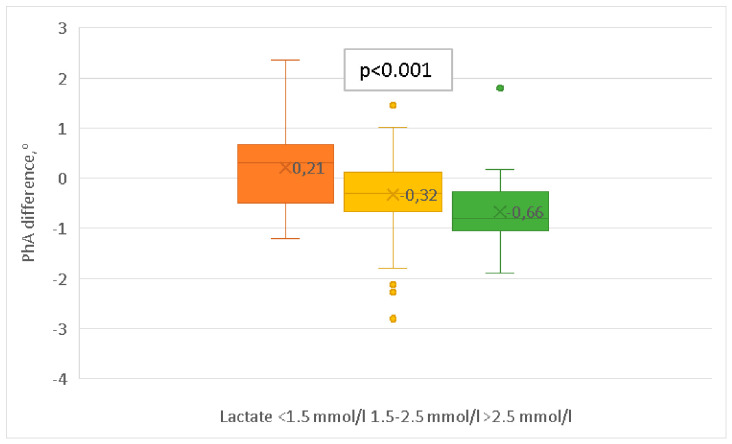
Change in PhA (Day 7 vs. Day 1) stratified by lactate levels. Boxplot showing change in PhA (in degrees), measured by BIA, grouped by lactate levels on Day 7 in the ICU. Statistically significant differences were observed between the <1.5 and >2.5 mmol/L lactate groups, as well as between the 1.5–2.5 mmol/L and >2.5 mmol/L lactate groups (*p* < 0.001).

To assess muscle loss as a potential indicator of tissue hypoperfusion, we categorised the patients into three groups based on their peak lactate concentration on Days 5 and 7: <1.5 mmol/L, 1.5–2.5 mmol/L, and >2.5 mmol/L. A statistically significant difference in the PhA and its percentage change was observed on Day 5 (*p* = 0.015 and *p* = 0.007) and Day 7 (*p* < 0.001 and *p* < 0.001), as illustrated in Figure 4 and Figure 5. However, no statistically significant differences were found in other BIA parameters or US muscle measurements.

Full data tables are provided in the Supplementary Materials Tables S3 and S4.

These findings highlight the nuanced physiological dynamics at play in critically ill patients, emphasising the pivotal role of fluid balance and inflammation in influencing the measurement of muscle deterioration.

## 4. Discussion

Evaluating muscle mass in the ICU presents challenges due to the lack of consistent methodology and the significant impact of fluid balance variations in critically ill patients. In our previous publication, we compared two methods—BIA and US—and found that US was the more suitable method for assessing muscle wasting during an ICU stay, while BIA failed to demonstrate comparable degree of muscle loss on Days 5 and 7, most likely due to the effects of positive fluid balance [18]. Nakanishi et al. published very similar findings: US was a suitable method for evaluating muscle loss, whereas BIA failed to yield accurate results under conditions of positive fluid balance [14]. There is a lack of studies investigating the impact of fluid balance on US measurements. Although the clinical importance of this effect—namely, the impact of fluid balance on muscle thickness measured by US—remains unclear, we conducted further analysis to explore it. In this article, we examined how positive fluid balance influences US-based diagnostics of muscle loss. Moreover, we investigated the association of the systemic inflammatory response and level of hypoperfusion with each of these methods (BIA and US).

In our study, we found that positive fluid balance has a statistically significant impact on BIA parameters: PhA and ECW/TBW significantly decreased, while FFM and SMM increased in response to positive fluid balance. Studies conducted with haemodialysis and intensive care patients have demonstrated similar results: positive fluid balance has a statistically significant impact on the same BIA parameters. Cleymaet et al. recently reported that BIA parameters are highly dependent on the hydration status, as well as on nutrition, age, and sex [16]. Based on these findings, it can be concluded that BIA is significantly influenced by the hydration status and may be useful for assessing fluid balance, but not for evaluating muscle wasting.

Regarding the use of US for assessing muscle wasting, no statistically significant effect of fluid balance category on US-derived muscle thickness changes was detected in our cohort. Hypothetically, this can be explained by changes in ICW and ECW content within the muscles. Studies conducted on healthy volunteers investigating the movement of water molecules within muscle tissue have demonstrated that under dehydration conditions, muscle volume decreases and subsequently restores under euvolemic conditions [11,13]. This suggests that the fluid status does not alter muscle structure or its US appearance. Another study involving older patients with sarcopenia showed that while the total water content within the muscle remains unchanged [12], it shifts from the intracellular to the extracellular compartment. Nevertheless, this shift can still be analysed using US because it does not influence the muscle structure itself. Taking these findings into account, US appeared less influenced by fluid balance than BIA-derived parameters and may therefore be a more reliable bedside method for assessing structural muscle wasting in this ICU cohort.

In the context of the inflammatory effect on muscle thickness measured by US, we found a statistically significant difference between the three patient groups categorised by CRP level (<100, 100–200 and >200) when comparing Day 5 to Day 7. By contrast, BIA values showed no significant differences between the groups. Multiple studies have shown an association between the severity of inflammation and the extent of muscle wasting [15,20,21]. Excessive activation of inflammatory pathways triggers downstream mechanisms of muscle loss: enhanced proteolysis, suppressed protein synthesis, and impaired muscle regeneration, ultimately resulting in skeletal muscle wasting [15]. Increased levels of pro-inflammatory cytokines are directly associated with reduced muscle mass [20,21]. In our study, US values reflected this same tendency.

Tissue perfusion, characterised by peak lactate levels (<1.5 mmol/L, 1.5–2.5 mmol/L, and >2.5 mmol/L), had no impact on US values. However, a statistically significant difference in the PhA and its percentage change was observed on both Day 5 and Day 7. As for other BIA parameters, lactate levels did not appear to influence them. These are highly interesting findings, which may be explained by the fact that the PhA is a marker of cell membrane integrity and cellular function, rather than simply muscle mass or volume. In critically ill patients, elevated lactate levels likely reflect impaired oxygen delivery and mitochondrial dysfunction—conditions that may result in cellular oedema, membrane damage, and altered capacitance—all of which directly affect the PhA. By contrast, US reflects structural rather than functional changes. This likely accounts for the absence of variation in US findings despite differing lactate concentrations.

This study builds upon our previous publication, where we first compared BIA and US in monitoring muscle wasting in critically ill patients [18]. While that work demonstrated the superiority of US over BIA, the potential effect of fluid balance on US measurements remained unexplored. The present analysis extends those findings in several important ways. We substantially increased the study cohort and additionally included COVID-19 patients, thereby enhancing the generalizability of our results. The methodological framework was strengthened by expanding the analysis to include cumulative fluid balance, systemic inflammation (CRP), tissue hypoperfusion (lactate), and additional BIA-derived parameters as factors potentially influencing the interpretation of US and BIA measurements. Taken together, this study provides a more comprehensive and clinically relevant assessment of the comparative utility of US and BIA in critically ill patients and suggests that US-derived muscle thickness measurements may be less influenced by fluid balance than BIA-derived parameters in this cohort.

### Limitations

This study has several limitations that should be acknowledged. It was a single-centre study conducted in two ICUs within the same tertiary hospital, which may limit the generalisability of the findings to other clinical settings with different patient populations, practices, or equipment. All measurements were performed by a single operator. Formal intra-rater reliability indices for ultrasound measurements, such as ICC or coefficient of variation, were not calculated in this study. Although all measurements were performed by a single trained investigator using a standardized protocol and repeated measurements were averaged, measurement error cannot be fully excluded. The study focused solely on muscle thickness changes and did not evaluate their association with functional outcomes such as muscle strength, mobility, or long-term recovery. The observational period was limited to 7 days, which may not adequately capture the full trajectory of muscle wasting, especially in patients with prolonged ICU stays. Another important limitation is the heterogeneity of the study population. The cohort included patients with different admission diagnoses, including COVID-19 pneumonia, acute pancreatitis, postoperative conditions, liver cirrhosis, sepsis, and other causes of critical illness. These conditions may be associated with different inflammatory profiles, fluid management strategies, capillary leak, nutritional trajectories, and rates of muscle wasting. Therefore, the relationships between fluid balance, inflammation, lactate levels, and US- or BIA-derived measurements may not be uniform across diagnostic subgroups. The sample size was insufficient to perform adequately powered subgroup analyses by admission diagnosis, and future studies should evaluate whether these findings differ across more homogeneous ICU populations. The presence of outliers in some measurements, combined with a modest sample size for subgroup analyses, may have influenced the statistical results and should be interpreted with caution. In our study, muscle wasting measurements were not compared with gold-standard imaging methods for ICU patients, such as CT. To date, only two studies have compared CT and US for monitoring muscle wasting in this population. The VALIDUM study [22] evaluated different muscle groups—CT measured muscle area at the L3 vertebral level, whereas US assessed the quadriceps—which may affect the results due to possible differences in wasting rates across muscle groups. Moreover, the second measurement in this study was performed in only a very small patient cohort, which is also a limitation of the study by Fetterplace et al. [10] Considering the current evidence, a direct comparison of CT and US measurements of muscle mass loss in ICU patients remains inconclusive. Further detailed studies are needed to validate or challenge the reliability of ultrasound for assessing muscle wasting in critically ill patients when compared with gold-standard techniques. The absence of statistically significant differences in US-derived muscle thickness changes between fluid balance groups should not be interpreted as evidence of equivalence. The analysis was not designed or powered as an equivalence study, and a type II error cannot be excluded, particularly in subgroup comparisons. In spite of these limitations, this is the largest study to date contributing significantly to knowledge in this field. Despite these limitations, the study provides clinically relevant evidence supporting the use of US as a bedside tool for monitoring muscle thickness changes in critically ill patients.

## 5. Conclusions

US reflects structural changes in muscle thickness and appeared to be a more reliable bedside method than BIA for monitoring muscle wasting in this ICU cohort. US-derived measurements were not significantly affected by fluid balance categories and showed sensitivity to inflammation-related muscle loss. In contrast, BIA-derived parameters were strongly influenced by hydration and perfusion status, suggesting that BIA may be more useful for evaluating fluid-related physiological changes than true structural muscle loss. Further studies comparing US with reference imaging methods and functional outcomes are needed to validate its role in ICU muscle monitoring.

## Figures and Tables

**Table 1:** Patient characteristics.

Parameter	Per-Protocol Sample (*n* = 143)
Age (years), mean (SD)	56.1 (13.9)
Body mass index (kg/m^2^), mean (SD)	31.3 (8.6)
**Admission type, *n* (%)**	
Medical	99 (69.2)
Surgical	42 (29.4)
Trauma	2 (1.4)
**Admission diagnosis, *n* (%)**	
Acute pancreatitis	23 (16.1)
Respiratory failure	60 (41.9)
COVID-19 pneumonia	42 (29.4)
Post-surgery	17 (11.9)
Liver cirrhosis	14 (9.8)
Sepsis,	8 (5.6)
Cardiovascular disease	7 (4.9)
Metabolic disease	2 (1.4)
Polytrauma	2 (1.4)
Other	10 (7.0)
APACHE II score, mean (SD)	16.6 (7.02)
SOFA score, Day 1, mean (SD)	6.7 (3.4)
NRS 2002 score, mean (SD)	3.9 (1.4)
Mechanical ventilation, *n* (%)	115.0 (80.4)
Duration (days), mean (SD)	14.21 (17.93)
Non-invasive ventilation, *n* (%)	8 (5.6)
Duration (days), mean (SD)	1.73 (2.11)
High-flow O_2_, *n* (%)	36 (25.2)
Duration (days), mean (SD)	5.51 (3.99)
Nutrition	
Kcal/kg/day, mean (SD)	16.6 (5.43)
Protein g/kg/day, mean (SD)	0.9 (0.34)
Renal replacement therapy, *n* (%)	53 (37.1)
CRP (mg/L), mean (SD)	277 (153)
Procalcitonin (µg/L), mean (SD)	26.7 (60.1)
Albumin (g/L), mean (SD)	23.6 (5.64)
Total fluid balance on Day 5 (mL), mean (SD)	1465 (4214)
Total fluid balance on Day 7 (mL), mean (SD)	825 (5238)
ICU LOS (days), mean (SD)	18.6 (16.9)
ICU mortality, *n* (%)	50 (35.0)

**Table 2:** Differences in BIA and US measurements (Day 5 vs. Day 1) across fluid balance groups.

	Negative (<−1000 mL)		Neutral (−1000 to 1000 mL)		Moderately Positive (1000 to 3000 mL)		Markedly Positive (>3000 mL)		
Parameters	*n*	Mean (SD)	*n*	Mean (SD)	*n*	Mean (SD)	*n*	Mean (SD)	*p*-Value
PhA ° change	37	0.24 (0.714) *^,^**^,^***	29	−0.21 (0.493) *	34	−0.44 (0.796) **	43	−0.47 (0.695) ***	<0.001
PhA % change	37	5.89 (16.792) *^,^**	29	−3.45 (13.308)	34	−8.04 (18.1) *	43	−11.82 (16.255) **	<0.001
FFM % change	37	−2.17 (4.762) *	29	1.67 (6.844)	34	2.91 (11.502)	43	6.29 (8.889) *	<0.001
FFMH % change	37	73.99 (0.629) *	29	74.3 (0.597)	34	74.4 (0.852)	43	74.74 (0.987) *	<0.001
SMM % change	37	−1.97 (5.015) *	29	1.31 (7.311)	34	2.39 (13.273)	43	9.7 (26.533) *	0.015
ICW/TBW change	37	0.6 (0.016) *^,^**	29	0.59 (0.013) ***	34	0.59 (0.018) *^,^****	43	0.58 (0.02) **^,^***^,^****	<0.001
ECW/TBW change	37	0.4 (0.016) *^,^**	29	0.41 (0.013) ***	34	0.41 (0.018) *^,^****	43	0.42 (0.02) **^,^***^,^****	<0.001
All muscles cm change	37	−0.79 (0.963)	29	−0.57 (1.07)	34	−0.51 (1.372)	43	−0.25 (1.34)	0.267
All muscles % change	37	−5.61 (7.748)	29	−5.05 (9.111)	34	−3.98 (12.084)	43	−0.38 (12.833)	0.134

BIA measurements: PhA ° change: phase angle change in degrees, FFM % change: fat-free mass percentage change, FFMH % change: TBW/FFM × 100%, representing the proportion of water content in the FFM, SMM % change: skeletal muscle mass percentage change. US measurement: All muscles change: change in muscle thickness measured by US. Statistically significant differences between fluid balance groups (Bonferroni-corrected pairwise comparisons): * *p* < 0.05 vs. negative. ** *p* < 0.05 vs. neutral. *** *p* < 0.05 vs. moderately positive. **** *p* < 0.05 vs. markedly positive.

**Table 3:** Differences in BIA and US measurements (Day 7 vs. Day 1) across fluid balance groups.

	Negative (<−1000 mL)		Neutral (−1000 to 1000 mL)		Moderately Positive (1000 to 3000 mL)		Markedly Positive (>3000 mL)		
Parameters	*n*	Mean (SD)	*n*	Mean (SD)	*n*	Mean (SD)	*n*	Mean (SD)	*p*-Value
PhA ° change	55	−0.02 (0.775) *	17	−0.2 (1.076)	30	−0.21 (0.726)	41	−0.59 (0.931) *	0.015
PhA % change	55	1.29 (18.035) *	17	−1.69 (25.744)	30	−5.3 (20.295)	41	−13.05 (19.246) *	0.007
FFM % change	55	−3.56 (8.897) *	17	−3.22 (7.893) **	30	2.84 (15.18)	41	5.65 (11.782) *^,^**	<0.001
FFMH % change	55	74.12 (0.916) *	17	75.08 (3.834)	30	73.7 (3.683)	41	75.14 (1.774) *	0.040
SMM % change	55	−4.04 (9.438) *^,^**	17	−3.68 (8.256)	30	6.83 (31.534) *	41	5.13 (11.494) **	0.009
ICW/TBW	55	0.6 (0.014)	17	0.57 (0.13)	30	0.59 (0.021)	41	0.58 (0.03)	0.057
ECW/TBW	55	0.4 (0.014) *	17	0.4 (0.016)	30	0.41 (0.021)	41	0.42 (0.03) *	0.004
All muscles cm change	55	−1.25 (1.326)	17	−0.89 (1.054)	30	−0.82 (1.139)	41	−0.82 (1.593)	0.360
All muscles % change	55	−9.41 (10.592)	17	−7.59 (8.944)	30	−6.97 (10.782)	41	−5.5 (14.788)	0.448

BIA measurements: PhA ° change: phase angle change in degrees, FFM % change: fat-free mass percentage change, FFMH % change: TBW/FFM × 100%, representing the proportion of water content in the FFM, SMM % change: skeletal muscle mass percentage change. US measurement: All muscles change: change in muscle thickness measured by US. Statistically significant differences between fluid balance groups (Bonferroni-corrected pairwise comparisons): * *p* < 0.05 vs. negative. ** *p* < 0.05 vs. neutral.

## Data Availability

The datasets generated and analysed during the current study are available from the corresponding author on reasonable request.

## Supplementary Materials

The following supporting information can be downloaded at https://www.mdpi.com/article/10.3390/nu18122019/s1. The following supporting information is available in the separate Supplementary Materials file: Figure S1: Comparison of percentage change in PhA and muscle thickness over time; Figure S2: Change in muscle thickness (Day 7 vs. Day 1) stratified by CRP levels; Figure S3: Percentage change in muscle thickness (Day 7 vs. Day 1) stratified by CRP levels; Table S1: Changes in BIA and muscle US measurements (Day 5 vs. Day 1) stratified by CRP levels; Table S2: Changes in BIA and muscle US measurements (Day 7 vs. Day 1) stratified by CRP levels; Table S3: Changes in BIA and muscle US measurements (Day 5 vs. Day 1) stratified by lactate levels; Table S4: Changes in BIA and muscle US measurements (Day 7 vs. Day 1) stratified by lactate levels.
