# Supplementary material for: Fluid Shifts and Muscle Loss in Critical Care: Accuracy of Ultrasound Versus Bioelectrical Impedance Analysis
Source: Nutrients. 2026 Jun 21;18(12):2019. doi: 10.3390/nu18122019 (PMC13304538; doi:10.3390/nu18122019)
Supplement: Supplementary file 1 [file nutrients-18-02019-s001.zip › nutrients-4364682-supplementary.pdf]

Supplementary Materials

Supplementary Figure S1. Comparison of percentage change in PhA and muscle thickness over time

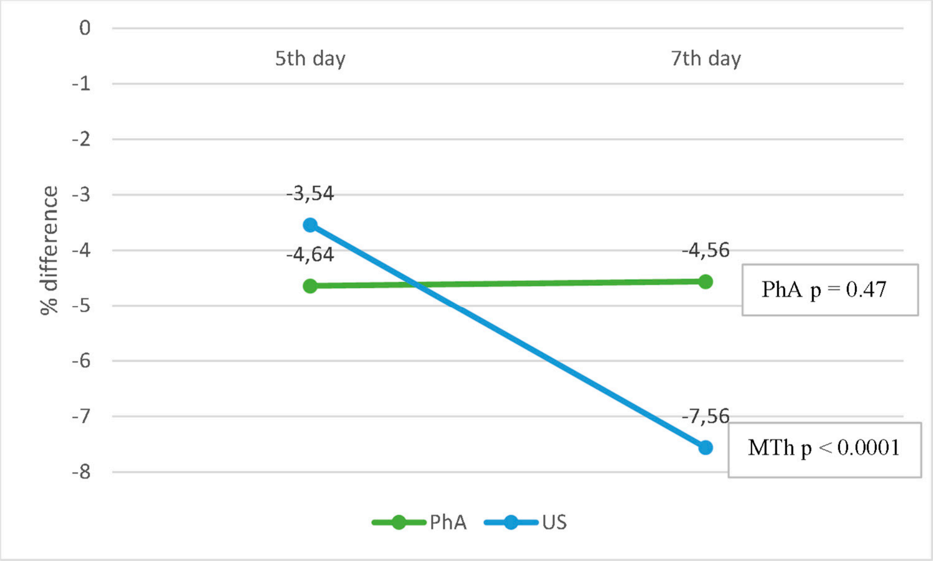

PhA, measured by BIA (percentage change), and muscle thickness (percentage change), measured by US, in time. Measurements on the first day were considered as baseline. There was no significant difference in PhA change during ICU treatment (p = 0.47), and a statistically significant difference was observed when comparing the changes in general muscle thickness (MTh) by measuring it with US on the fifth and seventh days (p < 0.001).

Supplementary Figure S2. Change in muscle thickness (Day 7 vs. Day 1) stratified by CRP levels

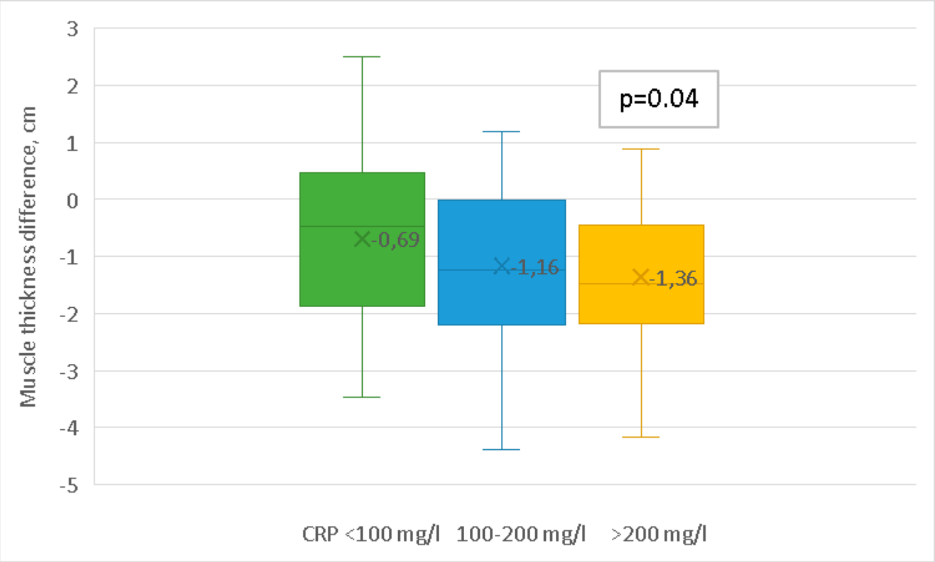

Boxplot showing absolute values (cm) change in muscle thickness measured by US, grouped according to CRP levels on Day 7. A statistically significant difference was observed between the <100 mg/L and >200 mg/L CRP groups (p = 0.04).

Supplementary Figure S3. Percentage change in muscle thickness (Day 7 vs. Day 1) stratified by CRP levels

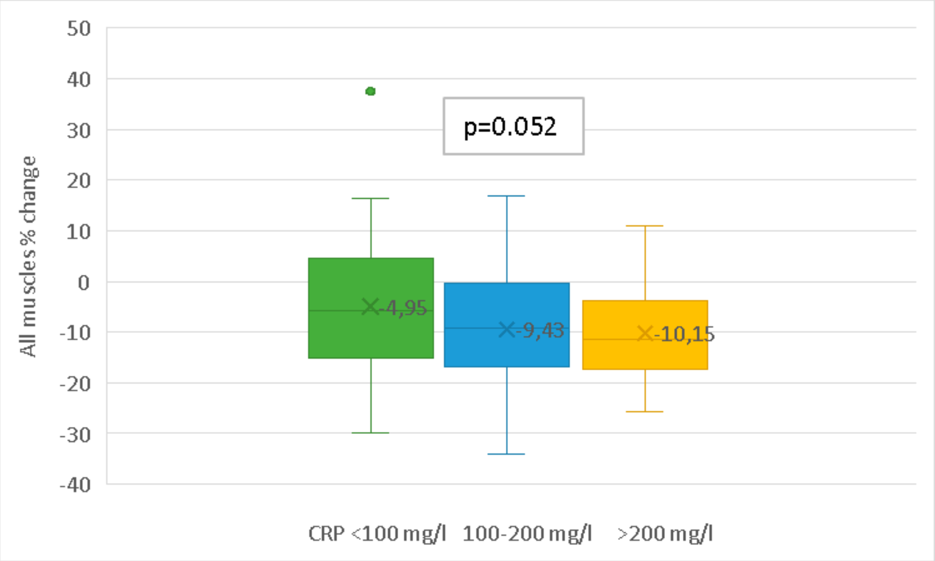

Boxplot showing percentage change in muscle thickness measured by US, grouped according to CRP levels on Day 7. A statistically nonsignificant difference was observed between the <100 mg/L and >200 mg/L CRP groups (p = 0.052).

Supplementary Table S1. Changes in BIA and muscle US measurements (Day 5 vs. Day 1) stratified by CRP levels

	<100 mg/L		100–200 mg/L		>200 mg/L		
**Parameters**	**n**	**Mean (SD)**	**n**	**Mean (SD)**	**n**	**Mean (SD)**	**p-value**
PhA ° change	66	−0.28 (0.543)	32	0.01 (0.813)	45	−0.32 (0.914)	0.124
PhA % change	66	−6.11 (13.491)	32	1.88 (19.656)	45	−7.11 (20.329)	0.055
FFM % change	66	2.06 (6.487)	32	3.12 (13.21)	45	2.27 (8.378)	0.858
SMM % change	66	1.62 (7.104)	32	3.09 (14.628)	45	5.73 (26.263)	0.457
ICW/TBW change	66	0.59 (0.019)	32	0.6 (0.02)	45	0.59 (0.02)	0.699
ECW/TBW change	66	0.41 (0.019)	32	0.4 (0.019)	45	0.41 (0.02)	0.686
All muscles cm change	66	−0.19 (1.193) *	32	−0.52 (1.298)	45	−1 (1.024) *	0.002
All muscles % change	66	−0.45 (12.183) *	32	−4.42 (10.536)	45	−7.43 (7.474) *	0.003

*Statistically significant difference between <100 mg/L and >200 mg/L CRP groups ($p < 0.05$)

Supplementary Table S2. Changes in BIA and muscle US measurements (Day 7 vs. Day 1) stratified by CRP levels

		<100 mg/L		100–200 mg/L		>200 mg/L	
**Parameters**	**n**	**Mean (SD)**	**n**	**Mean (SD)**	**n**	**Mean (SD)**	**p-value**
PhA ° change	65	−0.2 (0.821)	45	−0.24 (0.675)	33	−0.34 (1.187)	0.753
PhA % change	65	−4.16 (20.24)	45	−4.39 (16.896)	33	−5.56 (25.681)	0.949
FFM % change	65	−0.89 (9.853)	45	0.62 (13.193)	33	2.93 (13.353)	0.320
SMM % change	65	−1.32 (9.815)	45	2.57 (26.251)	33	3.06 (14.168)	0.378
ICW/TBW change	65	0.6 (0.019)	45	0.59 (0.021)	33	0.58 (0.097)	0.435
ECW/TBW change	65	0.4 (0.019)	45	0.41 (0.021)	33	0.4 (0.028)	0.105
All muscles cm change	65	−0.69 (1.413) *	45	−1.16 (1.345)	33	−1.36 (1.1) *	0.040
All muscles % change	65	−4.95 (12.89)	45	−9.43 (11.847)	33	−10.15 (8.293)	0.052

*Statistically significant difference between the <100 mg/L and >200 mg/L CRP groups ($p < 0.05$)

Supplementary Table S3. Changes in BIA and muscle US measurements (Day 5 vs. Day 1) stratified by lactate levels

	<1.5 mmol/L		1.5–2.5 mmol/L		>2.5 mmol/L		
**Parameters**	**n**	**Mean (SD)**	**n**	**Mean (SD)**	**n**	**Mean (SD)**	**p-value**
PhA ° change	45	−0.11 (0.864) *	67	−0.15 (0.716) **	31	−0.56 (0.499) *, **	0.015
PhA % change	45	−1.54 (19.715) *	67	−2.73 (17.548) **	31	−13.28 (10.572) *, **	0.007
FFM % change	45	3.21 (11.516)	67	1.53 (7.774)	31	2.94 (6.694)	0.573
SMM % change	45	2.99 (12.439)	67	3.76 (22.203)	31	2.48 (6.661)	0.936
ICW/TBW change	45	0.6 (0.022)	67	0.59 (0.019)	31	0.59 (0.015)	0.107
ECW/TBW change	45	0.4 (0.022)	67	0.41 (0.018)	31	0.41 (0.015)	0.101
All muscles cm change	45	−0.42 (1.271)	67	−0.57 (1.142)	31	−0.54 (1.304)	0.800
All muscles % change	45	−2.69 (10.004)	67	−3.94 (11.022)	31	−3.88 (12.131)	0.823

*Statistically significant difference between <1.5 mmol/L and >2.5 mmol/L lactate groups

**Statistically significant difference between 1.5–2.5 mmol/L and >2.5 mmol/L lactate groups

Supplementary Table S4. Changes in BIA and muscle US measurements (Day 7 vs. Day 1) stratified by lactate levels.

	<1.5 mmol/L		1.5–2.5 mmol/L		>2.5 mmol/L		
**Parameters**	**n**	**Mean (SD)**	**n**	**Mean (SD)**	**n**	**Mean (SD)**	**p-value**
PhA ° change	35	0.21 (0.876) *, **	86	−0.32 (0.834) *	22	−0.66 (0.75) **	<0.001
PhA % change	35	6.9 (23.43) *, **	86	−6.72 (18.037) *	22	−14.31 (17.62) **	<0.001
FFM % change	35	−2.31 (15.754)	86	1.02 (10.632)	22	2.73 (8.289)	0.234
SMM % change	35	2.88 (31.648)	86	−0.04 (9.657)	22	1.51 (7.891)	0.701
ICW/TBW change	35	0.6 (0.03)	86	0.59 (0.06)	22	0.59 (0.021)	0.211
ECW/TBW change	35	0.4 (0.03) *, **	86	0.41 (0.018) *	22	0.41 (0.02) **	0.013
All muscles cm change	35	−1.07 (1.155)	86	−1.03 (1.437)	22	−0.73 (1.29)	0.603
All muscles % change	35	−8.04 (9.678)	86	−7.86 (12.729)	22	−5.63 (11.499)	0.708

*Statistically significant difference between <1.5 mmol/L and 1.5–2.5 mmol/L lactate groups

**Statistically significant difference between <1.5 mmol/L and >2.5 mmol/L lactate groups
